# ‘Scared of going to the clinic’: Contextualising healthcare access for men who have sex with men, female sex workers and people who use drugs in two South African cities

**DOI:** 10.4102/sajhivmed.v19i1.701

**Published:** 2018-01-19

**Authors:** Zoe Duby, Busisiwe Nkosi, Andrew Scheibe, Ben Brown, Linda-Gail Bekker

**Affiliations:** 1 Desmond Tutu HIV Centre, Department of Medicine, University of Cape Town, South Africa

## Abstract

**Background:**

Men who have sex with men (MSM), sex workers (SW) and people who use drugs (PWUD) are at increased risk for HIV because of multiple socio-structural barriers and do not have adequate access to appropriate HIV prevention, diagnosis and treatment services.

**Objective:**

To examine the context of access to healthcare experienced by these three ‘Key Populations’, we conducted a qualitative study in two South African cities: Bloemfontein in the Free State province and Mafikeng in the North West province.

**Method:**

We carried out in-depth interviews to explore healthcare workers’ perceptions, beliefs and attitudes towards Key Populations. Focus group discussions were also conducted with members of Key Populations exploring their experiences of accessing healthcare.

**Results:**

Healthcare workers described their own attitudes towards Key Populations and demonstrated a lack of relevant knowledge, skills and training to manage the particular health needs and vulnerabilities facing Key Populations. Female SW, MSM and PWUD described their experiences of stigmatisation, and of being made to feel guilt, shame and a loss of dignity as a result of the discrimination by healthcare providers and other community. members. Our findings suggest that the uptake and effectiveness of health services amongst Key Populations in South Africa is limited by internalised stigma, reluctance to seek care, unwillingness to disclose risk behaviours to healthcare workers, combined with a lack of knowledge and understanding on the part of the broader community members, including healthcare workers.

**Conclusion:**

This research highlights the need to address the broader healthcare provision environment, improving alignment of policies and programming in order to strengthen provision of effective health services that people from Key Populations will be able to access.

## Introduction

### HIV amongst key populations in South Africa

Specific populations are disproportionately affected by, and more vulnerable to, HIV infection and its consequences, largely because of the criminalisation of certain behaviours, combined with societal and individual stigma and discrimination.^1,2,3^ For public health purposes, these socially marginalised groups are termed ‘Key Populations’. In this paper, we use this term to refer to people who use drugs (PWUD), sex workers (SW) and men who have sex with men (MSM). Structural and interpersonal barriers, including multiple forms of discrimination and exclusion experienced by these Key Populations at individual, community, health system and policy levels, impede access to healthcare, and the delivery of appropriate, non-discriminatory and non-judgemental services.^4^

The South African HIV epidemic is diverse, and within the generalised national epidemic there are several concentrated sub-epidemics.^5^ In 2015, an estimated 6.19 million people were living with HIV, and HIV prevalence amongst adults aged 15–49 years was estimated to be 16.6%.^6^ In 2015, approximately 153 000 individuals in South Africa made a living in the sex industry, and HIV prevalence amongst female SW in the three major metropolitan cities, Johannesburg, Cape Town and Durban, was estimated between 39.7% and 71.8%.^5,7^ No national MSM size estimate exists, and HIV prevalence has been estimated to range between 22.3% and 48.2% amongst MSM in the three largest metropolitan areas.^8,9^ The population of PWUD has not been quantified, but a modelling study estimated that in 2010 there were 67 000 people who inject drugs in South Africa.^10^ The only multi-city HIV prevalence survey amongst people who inject drugs, conducted in 2016, found an overall prevalence of 14%.^11^ In South Africa, laws criminalising sex work and drug use, as well as the broader social context of discrimination towards MSM, PWUD and SW, make it difficult to collect epidemiological data, as people are often reluctant to be counted as members of these populations for fear of arrest or discrimination.^12^

### Key population access to healthcare

Various structural factors limit the ability of Key Populations to access essential, appropriate and acceptable HIV prevention and treatment services.^13^ Globally, laws that criminalise behaviours such as drug use, sex work and same-sex relationships further marginalise Key Populations and perpetuate their exclusion from their communities and essential support services.^13^ Members of Key Populations commonly experience disapproval, rejection and suboptimal services in healthcare settings, to the point of their exclusion from the formal health system altogether.^12,14^ Stigma, a multilevel construct, ranging from individual to structural levels, has been conceptualised as a fundamental cause of health inequities amongst Key Populations.^15^ Structural or institutional discrimination refers to societal-level conditions such as practices and norms within institutions and social structures that deny rights or constrain the opportunities, resources and well-being of socially marginalised groups.^16^ Structural stigma has been defined as ‘societal-level conditions, cultural norms, and institutional policies that constrain the opportunities, resources, and wellbeing of the stigmatized’.^15^ In this study, we refer to the structural discrimination that Key Populations experience in the clinical setting, where human rights abuses and unethical treatment of Key Populations by healthcare providers are widespread.^13^

Even when Key Populations manage to access health services, those provided in the public sector health system in South Africa are often inappropriate, inadequate or insensitive to their particular needs. Examples include clinic opening hours that are unsuitable, particularly for SW; healthcare providers that take an ‘abstinence only’ approach to managing substance use; the lack of harm reduction services such as needle and syringe programmes; the absence of support groups targeted specifically at Key Populations; and the lack of standard routine risk assessment tools enquiring about penile–anal intercourse.^13,17,18^ Similarly, despite an early focus on preventing HIV and sexually transmitted infections (STIs) amongst SW, few scaled-up targeted interventions have been implemented in sex work settings, or amongst PWUD.^1^ Evidence shows that timely access to antiretroviral therapy (ART) and health services enabling viral suppression for Key Populations living with HIV is poor.^7^

While the South African Constitution does not discriminate against anyone on grounds of sexual orientation, in reality, gay men and other MSM continue to be stigmatised and discriminated largely because their behaviour deviates from social norms, and homoprejudice is widespread.^19,20^ A Key Population stakeholder consultation process conducted in South Africa in 2011 found that discrimination by healthcare workers towards MSM, SW and PWUD was a major barrier to accessing health services.^21^ In a study conducted in South Africa’s Gauteng province, 44% of the 487 lesbian, gay, bisexual or transgender study respondents reported having experienced heterosexism when accessing healthcare.^22^

Widespread discrimination, prejudice and moral-loading on the part of healthcare workers result in substandard healthcare provision and intensify Key Populations’ fear of seeking services.^2,16,23,24^ Fear of arrest and discrimination when disclosing particular practices to healthcare workers further limits Key Populations’ access to HIV prevention, treatment, care and support services.^25,26^ However, achieving a reduction in HIV incidence necessitates the adoption of approaches grounded in principles of human rights and inclusion, entailing the successful engagement of Key Populations in the health system to improve reach, uptake, access and utilisation of services, by creating enabling environments where non-discriminatory services are provided.^12,21^

### Research context: Free State and North West provinces

The study was conducted in two provincial capitals in South Africa: Bloemfontein (Free State) and Mafikeng (North West). These locations were selected based on lack of published research around these issues and potential scale-up of Key Population HIV prevention programmes in these areas at the time the research was completed. Both provinces share characteristics of being non-major metropolitan areas in rural provinces, with low population density compared to other provinces in South Africa. Free State contains 5.1% of the national population and North West contains 6.7%.^27^ North West is poorer than Free State, with 8.1% of the national poverty share, compared to 4.9% in Free State.^27^ No HIV prevalence data or population size estimates are available for MSM, SW or PWUD in these provinces. These provinces are characteristic of the public health system in South Africa beyond major metropolitan areas: no PWUD outreach programmes or harm reduction services exist,^28^ and availability of water-based condom-compatible lubricants is poor. Limited health services for MSM and SW have recently been provided through the Red Umbrella National Sex Work Programme.^14^

This article describes the findings from a qualitative baseline assessment of a study evaluating the ‘Integrated Key Populations Sensitivity Training Programme for Healthcare Workers in South Africa’.^29^ Here we present data relating to the context and Key Populations’ experiences of health service delivery in Bloemfontein and Mafikeng. Evaluation data of the training intervention itself will be presented in a separate paper.

## Methods

We used qualitative research methods to conduct a baseline assessment of the context of access to healthcare for MSM, SW and PWUD in Bloemfontein and Mafikeng. Data collection took place from March to August 2014. In-depth interviews (IDIs) were conducted with eight healthcare workers purposively sampled from four government health facilities in Bloemfontein and four in Mafikeng. Interviews enquired about healthcare workers’ knowledge, experiences and attitudes around service provision for Key Populations. In addition, six focus group discussions (FGDs) were conducted with 36 members of Key Populations (13 MSM, 13 PWUD and 10 female SW). Respondents were purposively sampled and recruited through organisations and networks in the respective cities. FGDs with MSM, PWUD and female SW explored experiences of stigma and discrimination in the community, and when accessing health services. All IDIs and FGDs were audio-recorded with permission of the respondents and were later transcribed and translated into English.

Qualitative data were analysed using an integrated approach, employing a deductive organising framework for code types, as determined by the content of the interview guides, combined with an inductive (ground-up) development of codes as they emerged from the data.^30^ A codebook was iteratively developed reflecting the key research questions, and the topics covered in the interview guides. The codebook was revised and modified throughout the coding process to ensure that it reflected the emerging themes. Qualitative data were coded and thematically analysed using NVivo 10 data analysis software by two analysts, with any identified discrepancies resolved through discussion until consensus was reached.

### Ethical consideration

Ethical approval for the study was granted by the Provincial Departments of Health in the Free State and North West provinces, as well as by the Human Subjects Research Ethics Committee at the University of Cape Town. All respondents provided written informed consent prior to their participation.

## Findings

Respondents in the IDIs and FGDs were similar across both provinces, as shown in Table 1. Qualitative data from IDIs and FGDs describing respondents’ experiences and perceptions are presented with direct quotations illustrating the key themes. The predominant narrative in the qualitative data pertained to the multiple forms of stigma and discrimination faced by PWUD, female SW and MSM at: (1) individual, (2) interpersonal (community) and (3) structural (health facility) levels. The data below are presented in relation to these three themes.

**TABLE 1:** Baseline respondent sample.

Population group	Total	Free State	North West
**SW**	**10**	**4**	**6**
Male	0	0	0
Female	10	4	6
Transgender	0	0	0
**PWUD**	**13**	**7**	**6**
Male	12	7	5
Female	1	0	1
Transgender	0	0	0
**MSM**	**13**	**8**	**5**
Male	13	8	5
Female	0	0	0
Transgender	0	0	0
**Healthcare worker**	**8**	**4**	**4**
Male	3	2	1
Female	5	2	3
Transgender	0	0	0

SW, sex workers; PWUD, people who use drugs; MSM, men who have sex with men.

### Individual internalised stigma

Stigma and discrimination experienced at the interpersonal and structural levels also resulted in feelings of shame and worthlessness, manifested as internalised stigma:

You don’t feel alright, as a person you know that is was not your intention to find yourself next to the road as a sex worker. It’s because you don’t have a job and there’s no way you can do anything because there are no jobs.’ [Female SW, FGD, Free State]

Key Population respondents seeking healthcare described their experiences of being made to feel guilt and shame by healthcare providers.

‘They (healthcare workers) make it like the sickness or the problem is your fault … that the issue you came with is your fault. They make you feel the guilt … you feel that whatever you are getting is deserved.’ [PWUD, FGD, Free State]

The negative health consequences, particularly health-seeking behaviours, related to internalised stigma were apparent in some of the narratives. Female SW respondents described situations in which nurses adopted a scolding tone, which caused feelings of embarrassment and shame, resulting in a reluctance to return to the clinic for treatment.

‘They (nurses) embarrass you … you end up telling yourself that you are no longer going to the clinic … they (nurses) make you uncomfortable, you become reluctant to go to the clinic.’ [Female SW, FGD, Free State]

Perceived and experienced stigma and discrimination within healthcare settings by Key Populations, particularly around sexual identity and sexual behaviour, led to internalised stigma which manifested in delayed care-seeking, travel to distant clinics and missed opportunities to receive appropriate services. One female SW respondent explained that she defaulted on her antiretroviral treatment because of the judgemental attitude of healthcare workers, which made her scared of going to the clinic.

‘There’s nowhere else I can go (for healthcare) … it has been three months since I last had my treatment. I take pills (ARVs) but because the Sisters don’t treat me well I have decided to stay without the treatment (implying HIV). This thing also makes me feel bad because I know that it is my life … They (nurses) just scold at you that you have come to irritate them. “We are not able to help you, go” … Nurses don’t treat us like people.’ [Female SW, FGD, Free State]

### Interpersonal discrimination

Key Population and healthcare worker respondents in both provinces described a context of discrimination at the interpersonal level. This interpersonal level discrimination in their communities was characterised by homoprejudice, discrimination and social exclusion experienced by Key Populations. The following quote from a healthcare worker in the North West alludes to the denial of the existence of homosexuality in the community, and the belief that homosexuality is a mental illness that should be prevented.

‘From where I come from what I have encountered is that in terms of homosexuality, there are no homosexual people. This thing of same-gender sex usually appears in psychotic patients, patients who are … ill. They are the ones you normally hear cases of them sleeping with one another, but in normal people it doesn’t happen … I don’t think that this facility can provide homosexuals with lubricant … Because we do not encourage that kind of sexual intercourse … The aim is to prevent intolerable behaviour, that’s our aim … trying to discourage all the inappropriate behaviours.’ [Healthcare worker, IDI, North West]

Another healthcare worker from the North West described how discrimination towards gay and lesbian people extends beyond healthcare facilities, and is evident in the broader community and religious institutions.

‘There is a lot of stigma around gays and lesbians. Gays don’t feel comfortable because of us in the community. Even in church gay people are not free because we don’t accept them the way they are.’ [Healthcare worker, IDI, North West]

In Free State, one healthcare worker attributed the discrimination towards MSM and gay men to the ignorance of community members.

‘(People) are ignorant … we know that it (MSM) is something that exists. People will tell you that the bible doesn’t agree, but it’s something that exists. People treat them in a different way like they are not human beings. If they get some sickness they say that they brought it on themselves which is not true. What they (community) need is information … they discriminate … There is a gay person on my street and they call him names, ‘isitabane’ and things like that, and it hurts him. That’s not right because he’s a person like us, he was created like that.’ [Healthcare worker, IDI, Free State]

A number of gay-identified MSM FGD respondents in Free State reported experiencing significant discrimination and rejection as a result of their sexual identity.

‘In my community I have a lot of people who don’t actually accept us as being gay … most of them are older people or the elderly, because they don’t understand what is being gay. They think it’s our choice to be this way, that we choose to be this way and we can change to being straight … also our own age groups give us a hell of a problem for being gay. They also think that whatever we do it’s our choice; we were not born this way. We are actually choosing this type of life, this type of sexuality for ourselves … in my community I have experienced a lot of problems … I have to actually go out of my community to feel accepted.’ [MSM, FGD, Free State]

All the Key Population respondents reported having experienced verbal abuse and being called derogatory names in the general community, and by healthcare workers specifically. Some of the gay-identified MSM respondents shared their experiences of homophobia and homoprejudice in their communities, manifested in actions such as being verbally abused, shunned and isolated. MSM described being called *isitabane* and *moffie*, offensive slang terms for homosexuals.

‘They call me a “moffie” … some of them will actually curse at me, give me some nasty words that I won’t even care to mention … Especially when you are in the taxis they show by their actions that “I don’t want to sit next to him” or “I don’t want him to touch me because he might infect me, he’s gay and everything”. That’s the type of things I actually experience in my community.’ [MSM, FGD, Free State]

Female SW were often called ‘*magosha*’ (prostitute), a derogatory term for SW in South African slang. The context of discrimination in the community was similar for PWUD, who were described by healthcare workers as being subject to stereotyping and assumptions of criminal and violent behaviour.

‘They think they (PWUD) are thugs, they mug people, they rape … If they don’t have money to buy drugs they will end up doing those things … so the community doesn’t accept them.’ [Healthcare worker, IDI, Free State]

People who use drugs also reported being called derogatory and offensive names and experienced a lack of trust from their families and friends because of their being perceived as untrustworthy and unreliable. Respondents from the PWUD focus groups described instances in which they had been labelled as being mentally unstable.

‘They treat me like a mad person, when I appear children start to run away. Children can run away because their parents tell them that this chap is mad.’ [PWUD, FGD, North West]

One theme that emerged in healthcare workers’ attitudes towards Key Populations related to a sense of blame and culpability, and the belief that SW, MSM and PWUD deserved to get HIV. Various Key Population respondents described the way in which nurses, often middle-aged women, adopt moralising judgemental tones when providing services to them.

‘There’s also discrimination whereby you find these old kinds of nurses who don’t have this knowledge about gays and lesbians … when you go to clinics and then maybe let’s say you have an STI or something. They then start calling you names, and saying “Guys don’t sleep with guys, why do you do that? … Boys don’t sleep together”.’ [MSM, FGD, Free State]

The narratives from healthcare workers themselves suggested that their moralising, judgemental and homoprejudicial attitudes are a result of religious conviction:

‘Those who don’t accept this thing of men sleeping with men, I would say it’s religious people mostly … the bible doesn’t accept that (homosexuality).’ [Healthcare worker, IDI, Free State]

Several of the healthcare workers cited their religious beliefs and Christian values in explanation of their own judgemental attitudes and lack of acceptance of behaviours such as sex work and same-sex partnerships. The following quotation illustrates a healthcare worker’s self-reflection on their own prejudicial attitudes and the need to resolve the conflict between religious beliefs and providing healthcare to those in need:

‘I am a Christian, I don’t believe in those things (homosexuality) actually. But because they are happening in a society that I am living in, then somewhere somehow it’s a dilemma I need to understand. Even if my religious belief does not allow me. People say this is wrong and we know that it’s wrong and it’s not accepted biblically. So people don’t accept them.’ [Healthcare worker, IDI, Free State]

### Structural discrimination

Several of the Key Population respondents narrated their personal experiences of violence, harassment and physical and sexual assault. Female SW respondents described their sense of powerlessness and their lack of recourse to bring incidents of discrimination or violence to the authorities because of the criminalisation of sex work; they explained that their lack of access to police protection enhances their vulnerability to violence. In addition to the fear of violence, respondents described their reluctance to disclose themselves as SW, MSM or PWUD to healthcare workers, or disclose their risk behaviours. As a consequence of this lack of disclosure, healthcare workers are often unaware of such patients’ risk behaviours, vulnerabilities or specific needs:

‘When it comes to health facilities … at the clinics, it’s about keeping it to yourself.’ [MSM, FGD, North West]

One barrier to disclosure to both police and healthcare workers was the lack of confidentiality:

‘They already know what you are … whether it’s a policeman or a nurse, will go to another colleague and say that you are from work (selling sex) … That one will also relay it (to colleagues) … Imagine getting that treatment here at hospital … we are even scared to go there because even if I get injured … they say that I was selling, I am a “magosha”.’ [Female SW, FGD, North West]

Many of the Key Population respondents described their personal experiences of having their confidentiality breached by nurses in government clinics:

‘Healthcare facilities … people who work there are not friendly. When you walk in they stare … (the nurses) will call their friends and tell them that you have an STI and they should come look. They tell you to undress. It will reach a point were you are scared of going to the clinic … they (nurses) call each other every time and about five of them would come. They would say come see, what is this thing? So that’s why some of us don’t go to these places, and that’s why some of us die of HIV/AIDS. That’s why.’ [MSM, FGD, Free State]

One SW respondent explained that as a consequence of the lack of confidentiality at the clinics, she chose to seek assistance instead from traditional herbalist healers to treat her STI symptoms.

‘I am scared of going to the clinic because we say these things in front of nurses … When she (nurse) leaves you in the room she goes out and tells people that ‘magosha’ are here to irritate them looking for condoms. ‘They are sick, the men they sleep with have given them sores’ … I have problems, I go to the clinic to present that problem, the Sister will leave me there and go talk. I can hear that this person is talking about me. I was even shy to leave the Sister’s room and go walk past those people (in waiting room) … I once had a problem with a very scary sore (STI) … Since I was scared of going to the clinic I took traditional Sotho herbs. There was no chance that I would go to the clinic because the Sisters talk about us … We are scared. We are terribly scared of nurses.’ [Female SW, FGD, Free State]

Notably, some Key Population respondents commented that healthcare workers are not homogeneous, and some provide services without discrimination or judgement:

‘They are not the same, there is one who will treat you fine … they are not the same; there are those who are fine and those who are not.’ [Female SW, FGD, Free State]

The sentiment that not all healthcare workers are unfriendly was echoed by some of the MSM respondents who explained that they had good relationships with healthcare workers and were open about being gay:

‘I have never had experiences like those … they are the friendliest towards gay people. Even when I am sick I go there and the service is okay unless they wait for me to leave and speak behind my back. People do that but I have not had such an experience.’ [MSM, FGD, North West]

In addition to healthcare workers displaying judgemental attitudes, many of the Key Population respondents shared the view that healthcare workers in government facilities are not equipped with the knowledge and skills to provide them with appropriate services:

‘The last time I went to the clinic there was this lady and she is very old. So she was busy writing and asking questions and stuff, then came the part where I had to take my clothes off. She was like where are you sick and then I had to tell her that my ‘other vagina’ (anus) is sick. She couldn’t understand. “What are you talking about?” “My A-S-S is sick”, and she was like “What happened? Did you have sex with your … (anus)?” I am like “yes, I am gay”.’ [MSM, FGD, Free State]

Structural barriers to accessing HIV-related commodities were also described by the Key Population respondents, such as the lack of access to clean injecting equipment for people who inject drugs. One PWUD explained that the only way for them to get clean needles is to steal them from the health facility:

‘What you do is that you wait in the Sister’s room and when they go out you just take a handful of needles … you steal them … I was too scared to ask (for needles), because I was scared I was going to get a reaction “What do you need them for?”. [PWUD, FGD, Free State]

Some healthcare workers expressed the opinion that providing an injecting drug user with clean needles would serve to encourage and condone the behaviour, and thus they were not prepared to do so:

‘I won’t encourage a person to commit a crime by giving them needles it means that I am encouraging them to continue with what they are doing.’ [Healthcare worker, IDI, Free State)

The physical environment of health facilities was also described as uninviting and non-inclusive of Key Populations, for example, the lack of informative and educational materials relating to Key Populations or their risk behaviours. As a result of the judgemental and discriminatory attitudes of public sector healthcare workers, and the non-conducive clinic environments, Key Population respondents expressed a preference for health services delivered through community and outreach-based programmes, or by the private sector.

## Discussion

These findings highlight the individual, interpersonal and structural barriers impeding access to healthcare for SWs, MSM and PWUD, and the delivery of health services in these two South African cities. Our findings support similar evidence from other cities in South Africa, such as Johannesburg, Tshwane, Durban, Pietermaritzburg and Cape Town.^23,31,32^ High levels of stigma and discrimination affecting Key Populations in communities and at health facilities were described by Key Population members who participated in the FGDs. Several healthcare workers described their own judgemental and moralising attitudes towards Key Populations.

This research illustrates prejudiced, discriminatory and judgemental views held by some healthcare workers in the areas in which this research was conducted, shaped by the prevalent social and religious norms and attitudes in their communities. Aligning with the experiences reported by members of the Key Populations, there was high self-reporting of moralistic and judgemental views by healthcare workers, coupled with a belief that healthcare professionals should provide ‘moral guidance’. These findings highlight the need for specific efforts to better align health service provision, especially in the public sector, with the South African Constitution, the *South African National Health Act*^33^ and the principles outlined in the Batho Pele White Paper,^34^ decreeing that healthcare should be impartial, non-judgemental and free of moral-loading. Additional Key Population policies that highlight the need for sensitisation training include the South African National Strategic Plan for HIV, TB and STIs (2017–2022), the South African National Sex Worker HIV Plan (2016–2019), the South African National LGBTI HIV Plan (2016–2019) and the Operational Guidelines for HIV, STIs and TB Programmes for Key Populations in South Africa (2012). In addition, these findings demonstrate the need for specific efforts to address judgemental attitudes amongst healthcare workers, providing them with the skills to provide the necessary support, counselling and services to Key Populations even when there is marked conflict with personally held moral views.

Key Population respondents in this study described their experiences of being made to feel guilt, shame and a loss of dignity as a result of the discrimination by healthcare providers. Evidence has shown that contexts of stigmatisation and discrimination may become internalised by individuals, manifesting at the individual level in psychological distress.^15^ ‘Internalised stigma’ or self-stigma refers to the cognitive, affective and behavioural processes in which stigmatised individuals engage in response to stigma-related stressors and the internalisation of negative societal attitudes about one’s social group.^15^ The South African HIV Stigma Index posited that internalised stigma is one of the most prevalent forms of stigma and can lead to reduced self-confidence, loss of motivation, withdrawal from social contact, avoidance of work- and health-based interactions, and abandonment of planning for the future.^35^ ‘Oppression illness’ refers to the negative emotional effects of internalising prejudice, social mistreatment and experienced stigma, including psychological or emotional harms, resulting in self-hatred, guilt and accepting blame for one’s suffering as just retribution for someone who does not deserve better treatment.^36^ Individual level stigma is associated with adverse health outcomes, devaluation of the self, poor self-regard, increased risk-taking behaviour, impeded health-seeking behaviour and negative health outcomes.^15,30,37,38^

Key Population respondents in this study described their reluctance to seek public sector healthcare and fear of disclosing their behaviours because of lack of confidentiality and concern around negative treatment from healthcare workers. These findings support other evidence showing that despite significant and sometimes urgent HIV treatment and care needs, many Key Populations do not access health services for several reasons, including fear of discrimination, humiliation, recrimination and breaches of confidentiality from community members, healthcare workers and the state.^15,25^ Fearing likely discrimination, possible legal consequences and even the refusal of services, SW, MSM and PWUD in these cities are reluctant to disclose risk behaviours and sexual practices to healthcare workers.^39^ The unwillingness of Key Populations has been shown to impede access to HIV testing and treatment services, resulting in poor adherence to medication, loss to follow-up, travel to more distant clinics and missed opportunities for appropriate service provision.^15,40^

The findings of this study provide insights into health service delivery for Key Populations in South Africa from the perspective of healthcare workers in these cities. As seen from some of the narratives of both healthcare workers and Key Population service users, it is evident that there is some level of engagement with Key Populations at health facilities; however, healthcare workers do not always have the necessary skills or support for this engagement. In this way, the lack of targeted training to inform health workers of the needs, health issues, strategies and interventions for Key Populations contributes to marginalisation and leaves healthcare workers ill-equipped to address health needs and perpetuates stigmatising and discriminating practices.^41^

The South African National Strategic Plan on HIV (2012–2016)^42^ recommended appropriate HIV prevention services for MSM, SW and PWUD and aimed to address discrimination. South African healthcare workers are constitutionally and ethically obliged to provide Key Populations with equitable, impartial care and treatment. However, as these findings demonstrate, discrimination of Key Populations by healthcare workers is a reality. Evidence has shown that efforts to effect structural change and the creation of ‘enabling’ environments through activities such as the training of healthcare workers to provide high-quality, non-judgemental services can be successful.^3,31^

One limitation of this research was the small sample size of respondents, and limited recruitment scope, meaning that the views expressed by respondents may not be representative of the broader communities; this limited generalisability is inherent in this type of research. Few PWUD participated in the study, and only one was female. Inclusion of more female PWUD, as well as male and transgender SW, would have provided additional insights into healthcare service experiences. It is possible that some of the nuance of respondents’ narratives may have been lost during the process of translation into English. Response bias may have been present in the respondents’ narratives, particularly amongst healthcare workers who may have expressed reserved attitudes out of concern for being judged by the researchers. Although this may have been the case, judgemental attitudes were described, suggesting that at least some of the healthcare workers openly disclosed their negative opinions towards Key Populations. Despite these limitations, the study findings provide insights with regard to the context of Key Populations and their access to healthcare in these settings.

### Recommendations

Sensitisation training of healthcare workers has been proven to be effective in reducing homoprejudice towards MSM and increasing knowledge and awareness of the specific vulnerabilities and health needs of Key Populations.^2,43^ Although sensitisation training on its own is likely to be insufficient, it is an essential step towards fostering enabling environments for effective health service provision for SW, MSM and PWUD. Sensitisation training needs to be combined with clinical competency training, which can improve the ability of healthcare workers to take appropriate medical histories, conduct relevant examinations, develop appropriate differential diagnosis and institute appropriate clinical management in a sensitive and appropriate manner.^38^ Clinical competency training is likely to be more effective amongst healthcare workers who have been ‘sensitised’, do not harbour prejudicial attitudes and are sensitive to the unique issues affecting and needs of SW, MSM and PWUD. Training interventions on their own are not sufficient to address barriers to accessing healthcare; they need to be complemented by demand generation and linkage to appropriate services, as well as the inclusion of mechanisms to monitor and address instances of stigma and discrimination.

Ongoing provision of resources, support and mentorship to enable the provision of sensitive, appropriate and effective health services is important, particularly as health workers face challenging situations. To ensure that Key Populations are willing and able to access the relevant healthcare, healthcare workers need to be non-judgemental, supportive, responsive and respectful and should understand the socio-environmental and health issues that Key Populations face. Providing healthcare workers in South Africa with sensitisation training and ongoing support would help to reduce discriminatory attitudes, increase compassion, encourage the use of non-judgemental language when working with persons who engage in behaviours that are often stigmatised and enable healthcare workers to carry out sexual risk assessments that do not assume heterosexuality. These changes, coupled with clinical competency training building on existing service provision practices, would enable healthcare workers to respond appropriately and effectively to the needs to Key Populations.^16,38^ Health service data collection tools and forms should reflect the heterogeneity of sexual and risk behaviour in a gender-neutral and non-judgemental manner, and requisite HIV prevention commodities should be available.

## Conclusion

Within the healthcare sector, stigma and discrimination take many forms at individual, interpersonal and structural levels. These findings highlight the need to address the policies that perpetuate the stigmatisation of Key Populations, increase efforts to reduce Key Population vulnerability to and risk of HIV infection and ensure equitable access to HIV testing, treatment and care. Better alignment of policies and programming is needed to strengthen the provision of effective health services that will reach Key Populations. Efforts towards increased HIV and STI testing and treatment, especially amongst Key Populations, are unlikely to be successful without addressing these issues. The need for sensitisation of healthcare workers through training and skills development is paramount to mitigate the discrimination of Key Populations, improving their ability to provide non-discriminatory, non-judgemental and appropriate health services to Key Populations, as well as the broader population. It is imperative that healthcare workers receive sensitisation training as part of an effort to ensure that the national HIV response is effective, and does not contravene the human rights and public health principles of freedom from discrimination and access to health services.
